# HE-DFNETS: A Novel Hybrid Deep Learning Architecture for the Prediction of Potential Fishing Zone Areas in Indian Ocean Using Remote Sensing Images

**DOI:** 10.1155/2022/5081541

**Published:** 2022-06-28

**Authors:** M. Sivasankari, R. Anandan, Fekadu Ashine Chamato

**Affiliations:** ^1^Department of CSE, Vels Institute of Science, Technology and Advanced Studies, Chennai 600117, India; ^2^Department of Chemical Engineering, College of Biological and Chemical Engineering, Addis Ababa Science and Technology University, Addis Ababa, Ethiopia

## Abstract

The Indian subcontinent is known for its larger coastline spanning, over 8100 km and is considered the habitat for many millions of people. The livelihood of their habitat is purely dependent upon the fishing activities. Often, the search for fish requires more time for catching and more resources, thus increasing the operational cost leading to low profitability. With the advent of artificial intelligence algorithms, designing intelligent algorithms for an effective prediction of fishing areas has reached new heights in terms of high accuracy (*A*_*cy*_) and less time. But still, predicting the location of potential fishing zones (PFZs) is always a daunting task. To reduce these issues, this work presented the novel hybrid prediction architecture of PFZs using remote sensing images. The proposed architecture integrates the deep convolutional layers and flitter bat optimized long short-term memory (FB-LSTM)-based recurrent neural networks (RNN). These convolutional layers are utilized to remove the various color features such as chlorophyll, sea surface temperature (SST), and GPS location from the satellite images, and FB-LTSM is utilized to predict the potential locations for fishing. The extensive experimentations are carried out utilizing the satellite data from Indian National Centre for Ocean Information Services (INCOIS) and implemented using TensorFlow 1.18 with Keras API. The performance metrics such as prediction *A*_*cy*_, precision (*P*_*scn*_), recall (*R*_*cl*_) or sensitivity (*S*_*ty*_), specificity (*S*_*fy*_), and *F*1-score and compared with other existing intelligent learning models. From our observations, the proposed architecture (99% prediction *A*_*cy*_) has outperformed the other existing algorithms and finds its best place in designing an intelligent system for better predicting of PFZs.

## 1. Introduction

The coastal oceanic atmosphere assumes a fundamental part in India's economy by excellence of their assets, useful living spaces and wide biodiversity. India has a long coastline of 7517 km incorporating islands which is a significant region both for investigation and misuse of common assets by the exclusive economic zone (EEZ) of 2.5 million km^2^. Marine fisheries area assumes a critical part in the economy as far as giving work to more than 14 million individuals and unfamiliar trade profit through export. The yearly marine fisheries creation in India is about 2.94 million tons against the harvestable capability of 3.93 million tons [1]. However, there is still a problem in finding the fishing areas that fishermen should visit [2, 3].

Prediction of fishing zone has been done utilizing the sea parameters derived either from satellite images or ground truth primary data [4, 5]. However, most of the research framework's utilized ocean graphic parameters such as chlorophyll and sea surface temperature (SST) feature for the prediction [6–8]. The prediction of fishing zones has now reached to new dimensions by the usage of machine and deep learning algorithms. Several algorithms such as long short-term memory (LSTM) [9], Markov models [10, 11], Naïve Bayes (NB) classifiers [12], support vector machines (SVM) [13, 14], and deep neural networks (DNN) [15–17] are used for prediction of fishery area based on different oceanographic parameters. However, an accurate prediction for PFZs still remains on the darker side of the research. To solve the aforementioned problem, this study proposes the novel hybrid model HE-DFNETS (hybrid ensemble DEEPFISHNETS) which integrates the double tier convolutional neural layers (DTCN) and flitter bat optimized LSTM (FOLSTM) for an efficient prediction of PFZs (PEZ) using remote sensing images. To the best of our knowledge, this work is the first of its kind utilized for the prediction of PEZ. The main contribution of the paper is as follows:

1. The ensembled convolutional layers are implemented to handle the different remote sensing images which comprise of sea surface temperature maps and sea surface chlorophyll (SSC) maps
2. Traditional training network is replaced with optimized long short-term memory (LSTM) for better performance.
3. Therefore, flitter bats are implemented to optimize the hyperparameters of the LSTM for a higher prediction rate.

## 2. Related Works

Rahul et al. introduced fishery information revelation dependent on help vector machines and fluffy principles to recognize fish stock, and the utilization of undersea innovation and GPS to develop programmed fishery examination frameworks are probably the most recent patterns being embraced around the world to improve, investigate, and grow the monetary fishing zones over the seas. This system helps for long-term forecast of the PFZ [18].

Su et al. utilized random forest (RF) and gradient boosting decision tree (GBDT) AI techniques to precisely infer saltiness inconsistency data in the worldwide subsurface and more profound sea (0–2000 m). As indicated by the outcomes, the RF model can well recover the SSA and beat the GBDT model. Besides, the exactness of the two models for the most part decline with profundity under 500 m [19].

Chacko et al. exhibit the helpful use of satellite information in the assessment of OHC (ocean heat content) with better spatial and temporal inclusion. This structure has assessed OHC700 in the Indian Ocean utilizing satellite-determined SST, (sea surface height anomalies) SSHA and OHC700 clim by utilizing the ANN procedure. The outcomes recommend the utility of the ANN strategy in assessing OHC700 with sensible exactness on a close continuous premise [20].

Wang et al. proposed an AI forecast strategy joined with wavelet change. This interaction utilizes information from upper sea perception floats put in the Arctic Ocean (AO.) to anticipate the sensor simple of chlorophyll-A (C.A.) in the upper expanse of the AO. A model joining SAE (stacked auto encoder) Bi (bidirectional) LSTM and wavelet change is proposed. From the experiment, these frameworks provide better results in regards to root mean square error (RMSE) and mean absolute error (MAE) [21].

Heyn et al. present a procedure to screen the ice condition continuously through assessment of boundaries that describe the appropriation of frame speed increases. It is shown how a Kullback–Leibler disparity measure can arrange ice condition among a bunch of pretrained conditions. The examination shows that the factual order techniques, planned by measure information, give steadier and more solid outcomes [22].

## 3. Proposed Framework: System Overview

**Figure 1:**
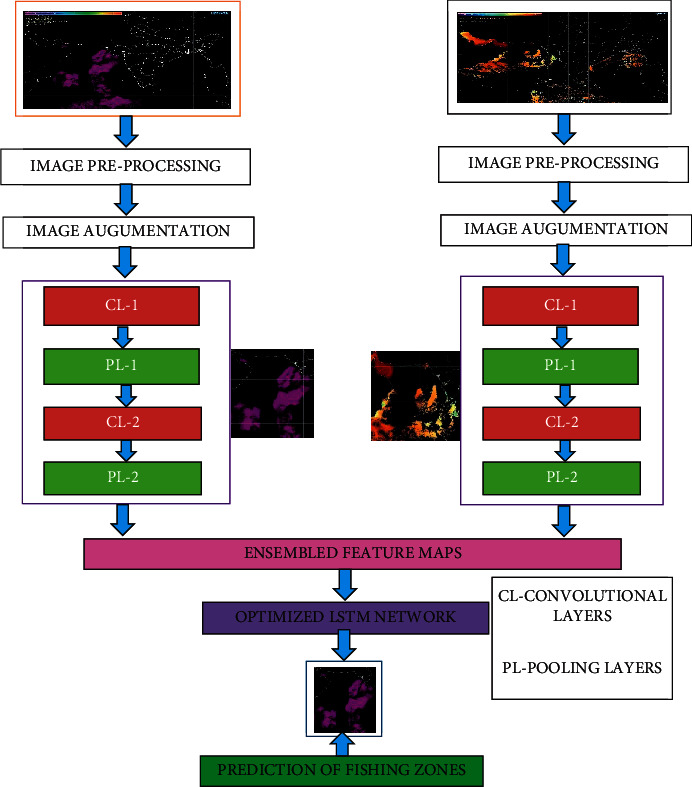
Proposed framework for the HE-DFNETS.

Figure 1 presents the architectural diagram for the proposed framework HE-DFNETS. The proposed HE-DFNETS architecture works on three different phases. The data collection unit collects from the satellites, image preprocessing and augmentation, segmentation with feature maps extractions using convolutional layers and finally flitter bat optimized LSTM networks.

### 3.1. Materials and Methods (Data Collection Unit)

A variable environmental database was accumulated consisting of SST, SSC, and GPS co-ordinates (latitudes and longitudes). Given their importance as environmental predictors of fishing zones [23], three variables are mostly used in modelling the proposed architecture. As mentioned in [24–26], SSC information gives data on sea's usefulness and are significant for recognizing fronts and vortexes that are not generally obvious in SST maps.  Table 1 shows the source of the different environmental satellite image data along with their characteristics and specification.

**Figure 2:**
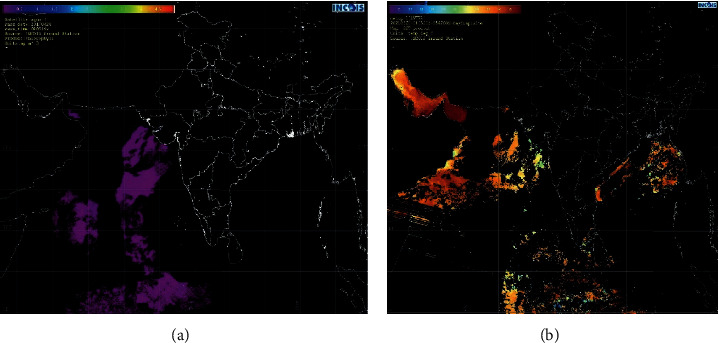
Sample image data used for training the network. (a) SSC maps. (b) SST maps.

Nearly 10 years of image data (Jan 1 2011 to April 2021) has been downloaded to train the proposed architecture.  Figure 2 shows the sample remote sensing images which represents the SSL and SSC parameters, respectively.

### 3.2. Image Preprocessing and Augmentation

The preprocessing technique is used to remove noise pixels, low-quality pixels which affects the prediction ratio. Image histogram methods are employed for enhancing the image quality even though the resolutions of obtained images are high resolutions.

### 3.3. Image Augmentation

To overcome the problem of overfitting, the image augmentation process is incorporated in the proposed framework. Though we have downloaded the ten years of remote sensing images, these image data are considered to be a limited quantity to train the network. Hence, data augmentation is adopted to tackle this problem. To perform the data augmentation, offline transformations [27] is applied on the series of each image which produces the large amount of newly corrected training image samples. The obtained augmented samples have the same correlations with original images, and this step is most widely used for preventing the overfitting problems as shown in Figure 1.

### 3.4. Proposed Network Training

This section details about the working mechanism of the proposed double-tier convolutional layers and flitter bat optimized LSTM.

#### 3.4.1. Proposed Ensemble Convolutional Layers

**Figure 3:**
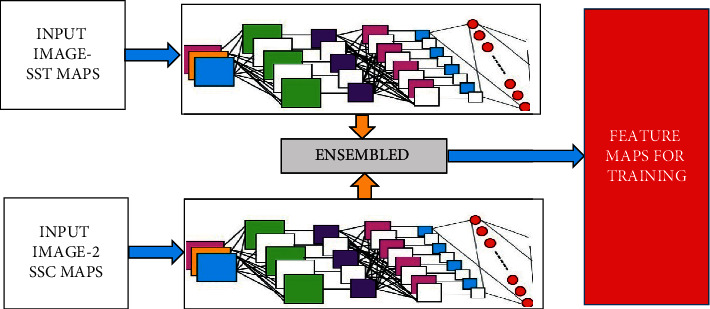
Double-tier ensembled CNN layers for an effective segmentation and feature extraction.

As mentioned in [28], the ensemble convolutional neural networks are used for an effective segmentation and feature extraction which is presented in Figure 3. The first-tier convolutional layers (Table 2) are used to segment the SSL remote sensing images in which the features are extracted and stored as separate feature maps. The similar fashion of convolutional layers (Table 2) are employed to extract the feature maps from the SSC maps.

Kim et al. [9] mainly focus on the assumption on temperature rise in the water by using the latest methods like “LSTM and deep learning” approach along with the “HWT” approach. So, the loss of all sea species can be prevented.

### 3.5. Optimized LSTM Training for Prediction

Then, the feature maps are extracted and ensembled for training the network. The proposed system replaces the traditional neural network training network with the flitter bat optimized long short-term memory. The proposed LSTM training network's working mechanism is presented in the preceding section.

#### 3.5.1. Hyperparameter Optimized LSTM Network

As mentioned in [17], though LSTM plays an important role in the prediction, performance of the network degrades when it handles larger datasets [27]. In the existing frameworks, there is computational complexity when the dataset scale gets increased. Motivated by this drawback, the proposed LSTM training must be aware of the computational complexity whose hyperparameters such as epochs, learning rate, and hidden layers are optimized by the bio-inspired flitter bat algorithms [29]. This approach will yield a better prediction rate compared to traditional network.

#### 3.5.2. Flitter Bat Optimized LSTM

Flitter bat algorithm is used to optimize the hyperparameters of the LSTM. The low complexity and less computational time of flitter bats than other bio-heuristic algorithms such as PSO and GA [30, 31] has inspired us to implement the flitter bats to optimize the hyperparameters of LSTM. In this case, no of epochs, learning rate, and hidden nodes are taken as the input bat population whereas the fitness function is calculated by

(1) $$\begin{array}{c} \text{fitness function }F\left(A\right)>\left\{A\left(n\right)-A\left(n+1\right)\right\}\;, \end{array}$$

where *A*(*n*) is the *A*_*cy*_ at initial stage, *A*(*n* + 1) is the *A*_*cy*_ at preceding section, and *n* is the number of iterations. The working mechanism of optimized LSTM is presented in Algorithm 1.

Finally, the fine-tuned hyperparameters in LSTM are used to predict the different PFZs effectively.

## 4. Experimental Setup

The proposed HE-DFNETS are implemented in TensorFlow 3.18 with Keras API which runs on “Windows PC10 with i7 CPU, 4 GB NVIDIA Geo-force GPU, 16 GB RAM and 2.5 GHZ”.

## 5. Performance Metrics and Evaluation

For the better classification, the images are resized to 256 × 256 × 3. Nearly 1, 06,700 image datasets were used for training.  Table 3 depicts the partitioned datasets utilized for both training and testing the network.

The hybrid combination of the CNN–FO-LSTM network is used in the proposed architecture whose hyperparameters are optimized by flitter bat algorithms. The sample images were used for training the proposed network. As the next step, the proposed architecture is tested with the images in which the ensembled convolutional layers extracts the image features and O-LSTM training networks classifies the appropriate categories. To prove the outstanding performance of proposed architecture, metrics such as “*A*_*cy*_, *S*_*ty*_, *S*_*fy*_, and *F*1-score” are calculated.  Table 4 shows proposed framework's validation parameters.

### 5.1. Results and Discussion

This section presents the significance of the proposed architecture over the other existing learning models in terms of various performance evaluations. The evaluation is carried out in tri-folded scenario. In the first scenario, prediction of PFZs for different areas are validated. Additionally, validation loss characteristics and receiver operating characteristics (ROC) are evaluated for the proposed architectures. In the next scenario, performance of the proposed architecture is compared with the other state-of-the-art learning models such as SVM [14], BILSTM [9], NB [12], gradient-boosted decision trees (GBDT) [21], artificial neural networks [17], and KNN-RF [32]. To overcome the imbalance problem, the proposed learning architecture is tested with random images.

#### 5.1.1. Scenario-I

In this evaluation, prediction *A*_*cy*_ of the proposed architecture is calculated for the Indian East Coastal Areas from the random dates from Jan 1 2021 to May 1 2021 along with the other performance metrics.

**Figure 4:**
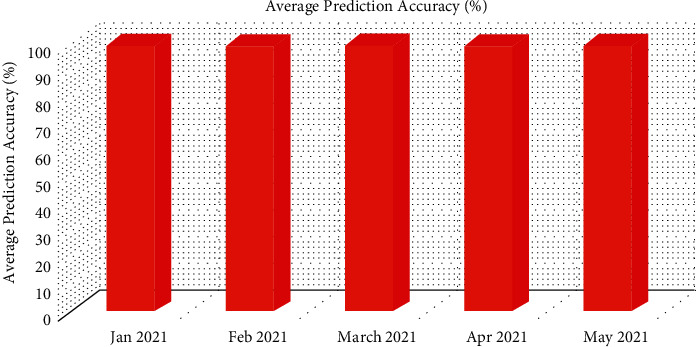
Average prediction *A*_*cy*_ of the proposed architecture in predicting the different fishing zones of the coastal parts of India.

Table 5 shows the prediction performance of the proposed architecture. The validation of the proposed architecture is done by random data sets in order to avoid the imbalance problems [33, 34]. From Table 5, it is found that the proposed architecture has predicted the PFZs accurately. The prediction *A*_*cy*_ of proposed architecture is shown in Figure 4. The integration of ensembled convolutional features and optimized LSTM training has yielded the 99% prediction *A*_*cy*_ which was observed from the month of January to May 2021.  Table 6 presents the average performance metrics such as “sensitivity, *P*_*scn*_, *S*_*fy*_, and F1-score”. The similar fashion of the performances (99% *S*_*ty*_, 99% *P*_*scn*_, 99% *S*_*fy*_, and 99% *F*1-score) has been found from Jan 2021 to May 2021.

#### 5.1.2. Scenario-II

Tables 7 and 8 give the comparative analysis of proposed framework vs. other existing frameworks.

Tables 7 and 8 show the comparative analysis between the proposed architecture and other learning models. It is found that integration of optimized LSTM with ensembled convolutional layers has maintained the average performance of 99% from the month of Jan 2021 to May 2021 and also it outperformed the other learning models such as ANN (55%), NB (60%), KNN (62%), RF (63.5%), GBDT (68.5%), SVM (70%), and BILSTM (78%), respectively.

**Figure 5:**
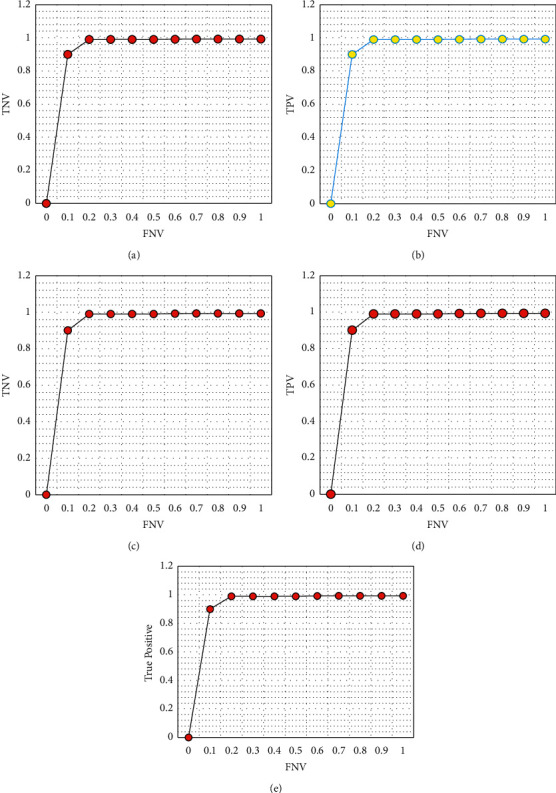
ROC characteristics. (a) ROC curve in predicting the PFZs based on South Tamil Nadu. (b) ROC curve in predicting the PFZs based on North Tamil Nadu. (c) ROC curve in predicting the PFZs in North Andhra Pradesh. (d) ROC curve in predicting the PFZs in Orissa region.

**Figure 6:**
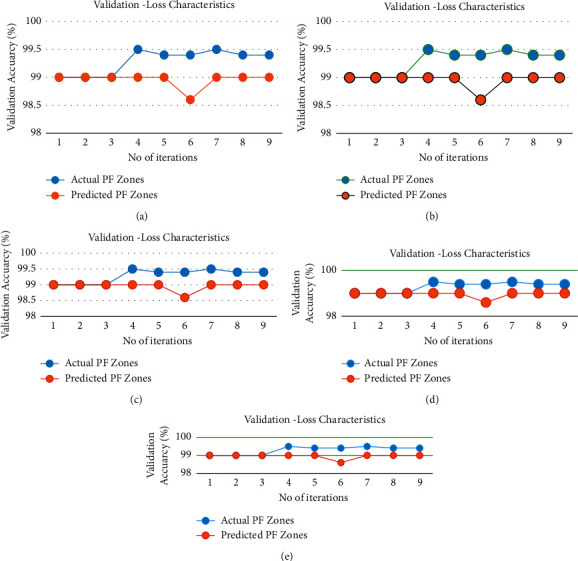
Average validation-loss characteristics of the proposed architecture. (a) Jan 2021. (b) Feb 2021. (c) March 2021. (d) April 2021. (e) May 2021.

Figure 5 shows the ROC characteristics of the proposed architecture for randomly chosen prediction zones.  Figure 6 shows the characteristics of validation loss of the proposed architecture. It is found in Figure 6 that loss is very less than 0.001 which is considered to be more suitable for the prediction of PFZs.

## 6. Conclusion

In this paper, a novel HE-DFNETS is proposed for the prediction of PFZs areas which can be used by the fisherman community. The proposed algorithm works on the principle of ensembled convolutional layers and replaces the traditional neural network training with the optimized LSTM network. In the proposed framework, the hyperparameters are optimized by the flitter bat optimization technique. The datasets include the satellite images which comprise SST and SSC along with GPS coordinates. The datasets were downloaded from https://incois.gov.in/portal/remotesensing/TERA_display.html. Extensive experimentations were accomplished using the above datasets, and validation metrics were calculated for different scenarios of the environment. The performance of the proposed architecture is validated randomly from the month of Jan 2021 to May 2021. It is found that the prediction *A*_*cy*_ is maintained uniformly to 99% for every month, and it outperforms the other state-of-the-art learning models. The above results show the promising performance of the proposed architecture in predicting the PFZs and can be utilized for the betterment of the fisherman's community.

### 6.1. Future Enhancement

Hence, the proposed architecture requires further enhancement in terms of reduced computational complexity.

## Figures and Tables

**Algorithm 1:**
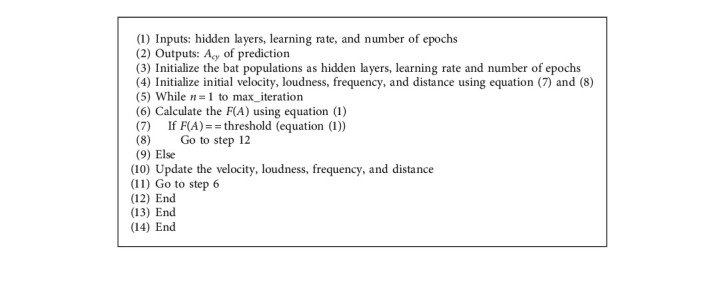
Algorithm 1 Optimized LSTM networks.

**Table 1:** Characteristics and specifications of satellite images collected with its source.

Environmental parameters	Source of data	Satellites	Resolutions (K)	Sensors
SSL parameters	https://incois.gov.in/portal/remotesensing/TERA_display.html	NOAA-17 METOP-1 METOP-2	4	AVHR MODIS
SSC parameters		MODIS		AQUA TERRA

**Table 2:** Parameters of CNN (Tier-1) used for segmentation and feature extraction OF SST maps and SSC maps.

Sl. no.	Layers used	Output size	Parameters
1	Convolution layer (CL-1)	256 × 256 × 32	564
2	Pooling layer (PL-1)	128 × 128 × 32	2
3	Convolution layer (CL-2)	128 × 128 × 64	2784
4	Pooling layer (PL-2)	64 × 64 × 128	0
5	Convolution layer (CL-3)	64 × 64 × 128	9673
6	Pooling layer (PL-3)	32 × 32 × 256	0
7	Convolution layer (CL-4)	32 × 32 × 128	167653
8	Pooling layer (PL-5)	16 × 16 × 256	0
9	Convolution layer (CL-4)	16 × 16 × 256	208960
10	Pooling layer (PL-5)	4 × 4 × 512	0

**Table 3:** Proposed framework's testing and training using image datasets.

Sl. no.	Total no. of images (after augmentation)	Training data (%)	Testing data (%)
01	1,06,700	70	30

**Table 4:** Mathematical expressions for the performance metrics' calculation.

Sl. no.	Validation parameters	Formulae
01	*A*_*cy*_	TPV+TNV/TPV+TNV+FPV+FNV
02	*S*_*ty*_ or recall	(TPV/TPV+FNV) × 100
03	*S*_*fy*_	TNV/TNV+FPV
04	*P*_*scn*_	TNV/TPV+FPV
05	*F*1-score	2.(Precison*∗*Recall/Precision+Recall)

TPV is true positive values, TNV is true negative values, FPV is false positive values, and FNV is false negative values.

**Table 5:** Predicted values of the different fishing zones by using proposed DEEPFISHNETS.

Date	Actual PFZ to be determined	Predicted PFZ by proposed architecture
Jan 2, 2021	North Tamil Nadu PF-zones	North Tamil Nadu PF-zones
Jan 15, 2021	West Bengal PF-zones	West Bengal PF-zones
Jan 25, 2021	Andaman and Nicobar PF-zones	Andaman and Nicobar PF-zones
Jan 31, 2021	Orissa PF-zones	Orissa PF-zones
Feb 4, 2021	North Tamil Nadu PF-zones	South Tamil Nadu PF-zones
Feb 14, 2021	North Andhra Pradesh PF-zones	North Andhra Pradesh PF-zones
Feb 28, 2021	South Andhra Pradesh PF-zones	South Andhra Pradesh PF-zones
March 1, 2021	North Tamil Nadu PF-zones	North Tamil Nadu PF-zones
March 10, 2021	West Bengal PF-zones	West Bengal PF-zones
March 23, 2021	Andaman and Nicobar PF-zones	Andaman and Nicobar PF-zones
March 31, 2021	South Tamil Nadu PF-zones	South Tamil Nadu PF-zones
April 7, 2021	Gujarat PF-zones	Gujarat PF-zones
April 14, 2021	West Bengal PF-zones	West Bengal PF-zones
April 20, 2021	Andaman and Nicobar PF-zones	Andaman and Nicobar PF-zones
April 23, 2021	Goa PF-zones	Goa PF-zones
April 30, 2021	North Tamil Nadu PF-zones	North Tamil Nadu PF-zones
May 2, 2021	Karnataka PF-zones	Karnataka PF-zones
May 15, 2021	South Tamil Nadu PF-zones	South Tamil Nadu PF-zones
May 20,2021	Orissa PF-zones	Orissa PF-zones

**Table 6:** Validation parameters (average) for the proposed architecture in predicting the PFZs.

Date	Performance metrics			
	*S*_*ty*_ (%)	*S*_*fy*_ (%)	*P*_*scn*_ (%)	*F*1-score (%)
Jan 2021	99.5	99	99	99
Feb 2021	99.5	99	99	99
March 2021	99.5	99	99	99
April 2021	99.5	99	99	99
May 2021	99.5	99	99	99

**Table 7:** Comparative analysis: proposed architecture vs. other state-of-the-art learning models in the months of Jan 2021-March 2021.

Algorithms	Performance metrics (%)				
	*A*_*cy*_ (%)	*S*_*ty*_ (%)	*S*_*fy*_ (%)	*P*_*scn*_ (%)	*F*1-score (%)
ANN	56	54.5	55	54%	55
NB	61	62	63	61.5%	62
KNN	63	62.4	62	61%	63
RF	64	62	64	61%	63
GBDT	67	66	65	62%	64
SVM	69	68.4	67.4	66.8%	66.30
BILSTM	78	75	76	775	76.5
DEEPFISHNETS	99	98.5	99	99%	99

**Table 8:** Comparative analysis between the performances of the proposed architecture and other state-of-the-art learning models in the months of April 2021-May 2021.

Algorithms	Performance metrics (%)				
	*A*_*cy*_ (%)	*S*_*ty*_ (%)	*S*_*fy*_ (%)	*P*_*scn*_ (%)	*F*1-score (%)
ANN	55	54.5	55	53	55
NB	60	62	64	60.5	62
KNN	62	63.4	64	61	63
RF	63.5	63	65	61	63
GBDT	68.5	67	64	62	64
SVM	70	69.4	66.4	66.8	66.30
BILSTM	78	75	75.6	77	76.5
DEEPFISHNETS	99	98.5	99	99	99

## Data Availability

The datasets used and/or analyzed during the current study are available from the corresponding author on reasonable request.
